# Widely applicable MATLAB routines for automated analysis of saccadic reaction times

**DOI:** 10.3758/s13428-014-0473-z

**Published:** 2014-05-02

**Authors:** Jukka M. Leppänen, Linda Forssman, Jussi Kaatiala, Santeri Yrttiaho, Sam Wass

**Affiliations:** 1 Infant Cognition laboratory, Tampere Center for Child Health Research, School of Medicine, University of Tampere, FIN-33014 Tampere, Finland; 2 Medical Research Council Cognition and Brain Sciences Unit, Cambridge, UK

**Keywords:** Vision, Attention, Oculomotor, Disengagement, Infant, Cognitive development, Saccadic reaction time

## Abstract

Saccadic reaction time (SRT) is a widely used dependent variable in eye-tracking studies of human cognition and its disorders. SRTs are also frequently measured in studies with special populations, such as infants and young children, who are limited in their ability to follow verbal instructions and remain in a stable position over time. In this article, we describe a library of MATLAB routines (Mathworks, Natick, MA) that are designed to (1) enable completely automated implementation of SRT analysis for multiple data sets and (2) cope with the unique challenges of analyzing SRTs from eye-tracking data collected from poorly cooperating participants. The library includes preprocessing and SRT analysis routines. The preprocessing routines (i.e., moving median filter and interpolation) are designed to remove technical artifacts and missing samples from raw eye-tracking data. The SRTs are detected by a simple algorithm that identifies the last point of gaze in the area of interest, but, critically, the extracted SRTs are further subjected to a number of postanalysis verification checks to exclude values contaminated by artifacts. Example analyses of data from 5- to 11-month-old infants demonstrated that SRTs extracted with the proposed routines were in high agreement with SRTs obtained manually from video records, robust against potential sources of artifact, and exhibited moderate to high test–retest stability. We propose that the present library has wide utility in standardizing and automating SRT-based cognitive testing in various populations. The MATLAB routines are open source and can be downloaded from http://www.uta.fi/med/icl/methods.html.

**Electronic supplementary material:**

The online version of this article (doi:10.3758/s13428-014-0473-z) contains supplementary material, which is available to authorized users.

A number of studies in nonhuman primates and humans have measured visuospatial orienting (i.e., rapid orientation of gaze and attention to a new stimulus appearing in a new spatial location) as a dependent variable to examine a variety of cognitive processes (Hutton, 2008; Johnston & Everling, 2008; Luna, Velanova, & Geier, 2008; McDowell, Dyckman, Austin, & Clementz, 2008). These include studies examining the development and neurocognitive bases of fundamental components of attention (Hunnius, 2007; Luna et al., 2008), the interactions between attentional and emotional processes (Fox, Russo, Bowles, & Dutton, 2001; Georgiou et al., 2005; Leppänen et al., 2011; Nakagawa & Sukigara, 2012), and the associations of core attention processes with higher-level cognitive (Franceschini, Gori, Ruffino, Pedrolli, & Facoetti, 2012; Rose, Feldman, & Jankowski, 2012) and emotion regulatory (Bar-Haim, 2010; Compton, 2000; Hakamata et al., 2010) processes. There is also emerging evidence from studies with special populations suggesting that deficits in visuospatial orienting may provide valuable markers for certain neurodevelopmental risk conditions, such as preterm birth (Hunnius, Geuze, Zweens, & Bos, 2008), autism spectrum disorders (Chawarska, Volkmar, & Klin, 2010; Elison et al., 2013; Elsabbagh et al., 2009), and neurocognitive deficits associated with fetal alcohol exposure (Green et al., 2009).

One of the most common ways to examine visuospatial orienting is to measure the latency of saccadic eye movements from the stimulus at fixation toward the location of the new stimulus in a new spatial location (i.e., saccadic reaction times, or SRTs). Various techniques have been used to analyze saccadic eye movements. Most often, manual coding of video recordings is performed to analyze participants’ eye movements (e.g., Haith, Hazan, & Goodman, 1988; Leppänen et al., 2011; Rose, Feldman, & Jankowski, 2004). Temporal resolutions of up to 50 Hz are available using these techniques (Elsabbagh et al., 2009); spatial resolution is low, but this is nonessential for tasks such as the present task, in which the aim is only to estimate the point at which the eyeball first deviates from the midline following a successful fixation. However, manual coding of video records is highly labor intensive, particularly with larger data sets, and prone to human error or biases. Another technique is to use electrooculography (EOG) to measure electrical potential changes resulting from the rotation of the eyes (e.g., Csibra, Tucker, & Johnson, 1998; Kemner, Verbaten, Cuperus, Camfferman, & van Engeland, 1998). The temporal resolution of these techniques is high. Again, spatial resolution is low, but this is nonessential for present purposes. However, these techniques involve the administration of electrodes, which can be distressing for some participants, perturbing data and causing data loss.

In the last decade, there has been a rapid increase in the use of new corneal reflection eye-tracking techniques to measure eye movements, particularly in studies involving special populations such as infants and young children. In essence, eye tracking is a noninvasive technology that has the advantage over other techniques in that it offers the possibility for automated acquisition and analysis of eye movements at a high spatial and temporal resolution, is less labor intensive, and minimizes the possibility of human error or biases (Aslin, 2012; Elison et al., 2013; Gredebäck, Johnson, & von Hofsten, 2009; Morgante, Zolfaghari, & Johnson, 2012; Oakes, 2012). A particular advantage of eye-tracking technologies for researchers measuring SRTs as the dependent variable is that the metrics of interest can be extracted from the gaze data by using a simple, automated routine (e.g., an algorithm that identifies the time point at which the gaze leaves or enters an area of interest). Recent studies have, however, demonstrated that the practice of such analyses is complicated by several limitations in the temporal and spatial accuracy of current eye-tracking technologies, especially when used with poorly cooperating participants (Frank, Vul, & Saxe, 2012; Morgante et al., 2012; Shic, Chawarska, & Scassellati, 2008a, 2008b; Wass, Smith, & Johnson, 2014). Similar discussions are ongoing in the adult literature (Blignaut & Wium, 2014; Holmqvist et al., 2011; Nyström, Andersson, Holmqvist, & Weijer, 2013).

Recently we have investigated two aspects of eyetracker data accuracy and quality that appear to be particularly variable in studies with poorly cooperating participants—namely, precision, the consistency in the reported position of gaze between samples, and robustness, how broken or fragmented contact with the tracker is during recording (Wass, Forssman, & Leppänen, 2014). Our study showed that, if widely used analytical techniques are followed, a number of key dependent variables in eye-tracking experiments can be disrupted by between- and within-subjects variations in these aspects of data quality. For example, we found that less precise data can appear to suggest a reduced likelihood to look at a narrowly defined area of interest (such as the eyes in a face, relative to the mouth). We also found that less robust data can appear to manifest as shorter fixation durations and shorter first look/visit duration. Finally, we found that less robust tracking may manifest as longer SRTs (e.g., time to first fixation). Together, these results suggest the importance of taking steps to control for data quality before performing final analyses.

Given the obvious potential of the eye-tracking technology for SRT analysis (and the widespread use of SRTs in behavioral studies), we set out a project to examine whether automated analyses of SRTs from eye-tracking data can be implemented in a way that is robust against variations in data quality and potential sources of artifacts. A further goal of the project was to develop techniques that could be used as a standardized method in a number of SRT paradigms and studies, including studies with poorly cooperating participants. The project resulted in a library of MATLAB (Mathworks, Natick, MA) routines for preprocessing and analysis of SRTs from eye-tracking data (http://www.uta.fi/med/icl/methods.html). The preprocessing routines consist of data interpolation and median filtering function that are applied to raw eye tracking to cope with problems in data quality. The SRT analyses routines include algorithms for detecting saccadic eye movements and several postanalysis “check” functions that enable the user to automatically identify (and reject) SRTs that have a high likelihood of being inaccurate or contaminated by artifacts. To test the proposed routines, we used data from human infants to compare the SRTs obtained by the automated scripts with SRTs obtained manually from video records, examined the robustness of the analyses against indicators of data quality (precision and robustness) and accuracy of calibration, and analyzed the test–retest stability of the SRTs over repeated testing of the same infants from 5 to 7 months of age and from 9 to 11 months of age.

## Method

### Typical SRT paradigms

**Fig. 1:**
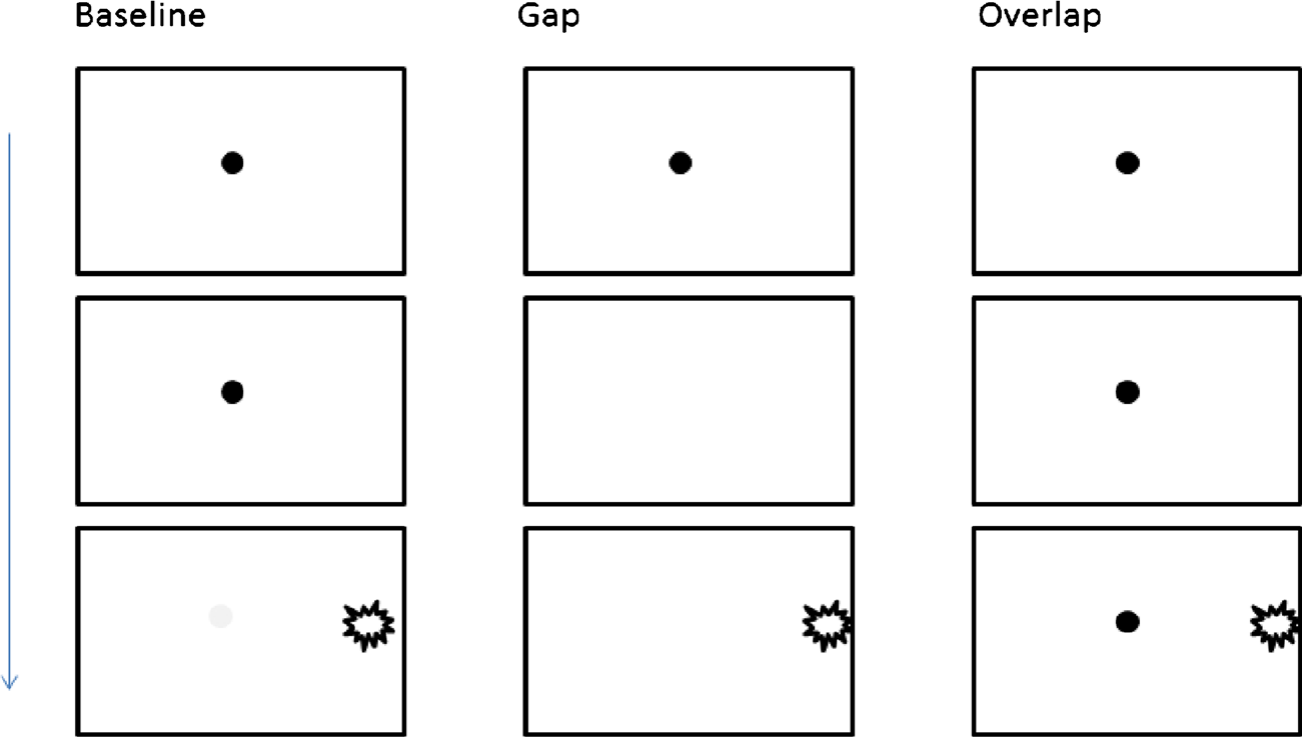
An illustration of the paradigm used to measure saccadic reaction times and visuospatial orienting. In the “Baseline” condition, the first (central) stimulus is extinguished upon the onset of the second (lateral) stimulus. In the “Gap” condition, the first stimulus is extinguished before the onset of the second stimulus. In the “Overlap” condition, the first stimulus remains visible throughout the trial. The overlap condition differs from the first two in requiring an active process of attention disengagement from the stimulus at fixation prior to the movement of the point of gaze to the new stimulus and, therefore, saccadic reaction times in this condition are typically longer

A widely-used paradigm for measuring SRTs includes the presentation of two stimuli with a slight (e.g., 1,000 ms) onset asynchrony (Aslin & Salapatek, 1975; Csibra et al., 1998; Elison et al., 2013; Elsabbagh et al., 2009; Hood, 1995; Hunnius, 2007; Hunnius, Geuze, & van Geert, 2006; Johnson, Posner, & Rothbart, 1991; Scerif et al., 2005). Typically, the first stimulus is presented at the center of the stimulus display, and the second laterally to the left or right periphery. There are several variations of the paradigm that place varying demands for attention (see Fig. 1 for examples of the typical variations), but the SRTs are invariably measured as the latency at which the point of gaze moves from the location of the first stimulus to the location of the second stimulus (i.e., leaves the area of the first stimulus area or, alternatively, enters the area of the second stimulus).

The SRT paradigms used with infants are similar to those used in older (verbal) children and adults, with the exception that infant paradigms rely on infants’ spontaneous tendency to orient to new stimuli, whereas older children and adults are typically given verbal instructions to orient to the lateral stimuli (Green et al., 2009; Luna et al., 2008; McDowell et al., 2008; Müri & Nyffeler, 2008). This specific aspect of infant paradigms is important, since infants’ spontaneous saccadic eye movements appear to depend significantly on the properties of the attention-grabbing stimulus. For example, studies using static geometric shapes as lateral stimuli have shown a steady reduction in visuospatial orienting to the lateral stimulus after repeated trials (Leppänen et al., 2011), possibly reflecting simple habituation of orienting to the peripheral stimulus or, alternatively, infants’ voluntary inhibition of repeated attention shifts to the peripheral stimulus (Holmboe, Fearon, Csibra, Tucker, & Johnson, 2008). Our unpublished data (shown in Supplementary Fig. 1) suggest that the attention shift rate remains reasonably steady when the peripheral stimulus is changed from a static picture to a dynamic animation, and the onset of the animation is programmed to be contingent upon eye gaze entering the target area (i.e., the animation starts to play when the infant’s point of gaze reaches the area of the animation). Such gaze-contingent features can be programmed in most software integrated with eyetrackers (for example, in E-Prime software or Psychtoolbox and Talk2Tobii toolbox or the Tobii Analytics SDK for interfacing with Tobii eye-tracking systems, Tobii Technology, Stockholm, Sweden).

### Analysis of SRTs from eye-tracking data

#### Raw data

**Fig. 2:**
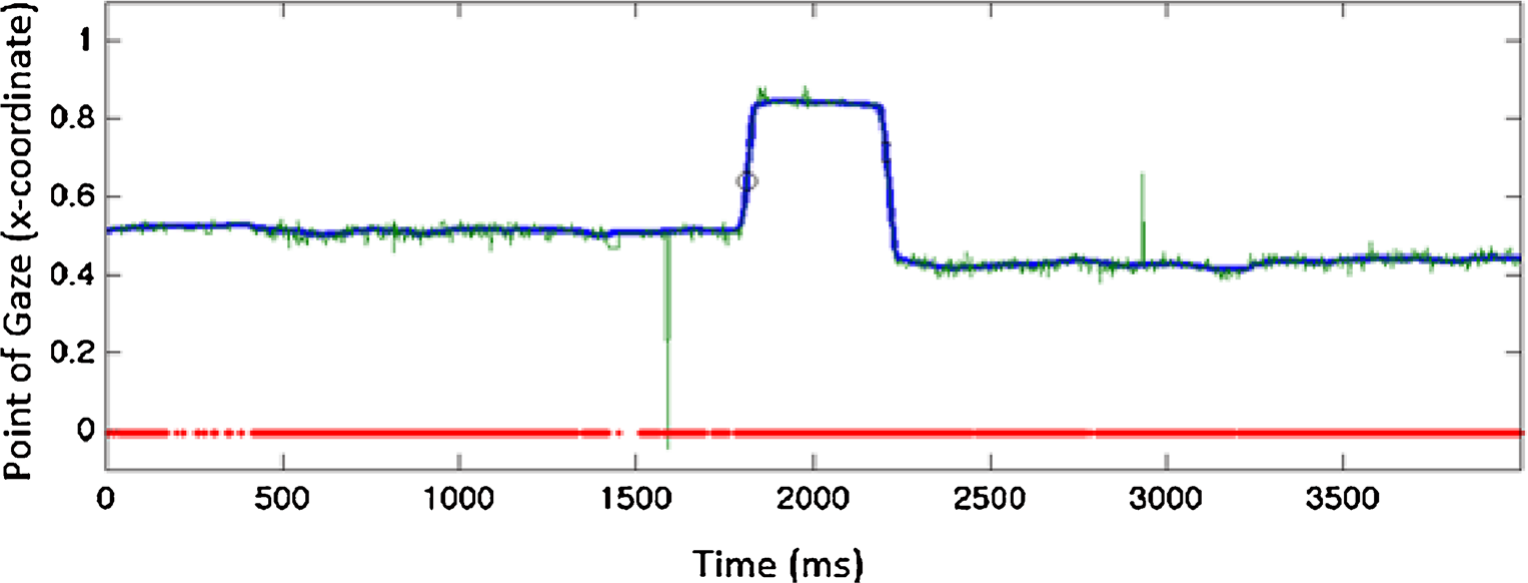
*X*-coordinates of gaze location as a function of time for one trial of a 7-month-old infant. The data were recorded in a paradigm involving a central stimulus (a picture of a face or a facelike pattern) and a lateral stimulus (a geometric shape). The lateral stimulus was presented at 1,000 ms. Raw values for the point of gaze are shown by the narrow green line, and interpolated and median-filtered values by the thick blue line. Saccade is indicated by an abrupt change in the *x*-coordinates ~1,700 ms from the start and is measured as the last sample before the point of gaze leaves the area of the first stimulus (indicated by an open circle)

Most eye-tracking software provide raw gaze data, with the following variables that are critical for the present analyses: (1) *x*- and *y*-coordinates for the point of gaze on the screen (separately for each eye), sampled at the specified temporal resolution (60–300 Hz in most eyetrackers used with infants), (2) time stamps for each data sample (e.g.,“Tobii Eye Tracking or “TETTime” provides the time stamps at microsecond accuracy), (3) information about the “validity” indicating the reliability of tracking at each time point (e.g., Tobii TX300 uses codes 0–4, with codes 0 or 1 typically considered to indicate technically reliable gaze tracking), and (4) additional time stamps to provide exact synchronization between eye tracking and stimulus presentation (e.g., a column specifying the stimulus that is currently on screen). The *x*-coordinates of the gaze location for one overlap SRT trial of a 7-month-old participant are shown in Fig. 2 (the *y*-coordinates were omitted from the visualization because these tend to remain relatively stable across time in paradigms in which the first and the second stimuli are aligned on the vertical axis). The visualization illustrates two common characteristics of eye-tracking data collected from infants (Wass et al., 2013, 2014). First, the raw data includes occasional periods of missing or unreliable data (shows as gaps in the thick red line at the *y* = 0). Second, the point of gaze undergoes constant fluctuation at periods of fixation (a problem known as low precision of eye tracking). The visualization further shows that the *x*-coordinates show an abrupt change at the time of the saccade.

#### Preprocessing: interpolation and filtering

The attrition rate in infant eye-tracking studies can be relatively high due to fragmented or low-quality data caused by, for example, poor calibration, excessive movements, or lapses in attention. Analyses presented in the Supplementary Results show that in eyetracker data obtained from typical 12-month-olds under optimum laboratory testing conditions, 17.9 % of all available data samples were missing and 62 % of all usable data segments obtained were of under 1 s in duration (see Supplementary Fig. S2). To address this problem, we implemented an *interpolation* routine that identifies the last recorded *x*- and *y*-coordinates for one or both of the eyes and continues these values forward until the data come back online (Wass et al., 2013). In our approach, the interpolation routine is applied to all periods of missing data regardless of their duration, but importantly, the user should specify a postanalysis check function to identify trials that were contaminated by extensive interpolations (i.e., unreliable trials), as described below.

Another common problem with eye-tracking data is abrupt changes in the point of gaze that are attributable to technical artifacts. For example, in the data shown in Fig. 2, the *x*-coordinate changes abruptly from ~ .5 to 0 (equaling a 23° change in visual angle) for the duration of a few milliseconds at around 1,550 ms poststimulus. Removing such spikes from the data is critical to avoid false SRTs occurring when a spike crosses the AOI border during the window of interest (Fig. 2). To remove this artifact, we implemented a moving *median filter*. The length of the median filter can be specified by the user, and both ends of the analysis period are truncated with the first or last available sample to enable the filter to be applied for the whole analysis period.

#### Analysis of SRTs

The SRTs are determined as the last data point in the first stimulus area, preceding the transition of the gaze to the direction of the second stimulus area. The areas of interest for the first and second stimulus can be adjusted by the user. The SRT for the example data in Fig. 2 is shown as a small open circle superimposed on the raw and preprocessed gaze data. If no gaze shift is recorded within the specified analysis period (e.g., the point of gaze does not move from the first stimulus to the second stimulus within the specified time window), the value of the SRT is determined as the last data point of the analysis window (e.g., 1,000 ms for an analysis window ranging from 150 to 1,000 ms poststimulus). As we explain below, condition and subject-specific mean SRTs can be calculated on the basis of trials with gaze-shifts only or by using an index that combines data from all trials (i.e., trials with and without gaze shifts).

#### Postanalysis verification checks

Postanalysis verification checks were implemented to eliminate unreliable SRTs from the data. First, the user can set *a minimum and a maximum for the duration of the first and second stimuli* to eliminate trials where the actual duration of gaze data for a trial deviates from the set duration of the trial (i.e., the eyetracker fails to record for the entire duration of the trial, or the software used for stimulus presentation fails to present the stimulus for the required duration). In our experience, such deviations exist but are fortunately very rare in the software interfacing with Tobii eyetrackers. Second, the user can set an *upper limit for the interpolated segments* (e.g., 200 ms) to eliminate the possibility that real SRTs (e.g., central–lateral–central gaze transitions as illustrated in Fig. 2) are missed due to interpolation, and erroneously determined as maintenance of the gaze within the area of interest. Third, a *border violation* check is included to detect transitions between areas of interest that were missed during interpolated data segments. The rationale behind this function is that interpolating segments of missing data is acceptable if the gaze remained within the area of interest throughout the interpolated period (assuming that the longest accepted interpolated segment was too short to enable quick gaze shifts between areas of interest during the period of interpolation). However, if the area changes during the missing data segment, then a gaze shift has taken place during the missing data segment, and the disengagement time from the original area to the new area cannot be reliably determined. In these cases, border violation is noted, and the SRT is excluded from the final data. Finally, a user-defined criterion is used to detect trials without *minimum required fixation time for the first area of interest* prior to saccade. This function ensures that trials during which the gaze was not sufficiently long in the area of interest for the first stimulus prior to the saccade (e.g., because the participant did not pay attention or looked away from the first stimulus) are eliminated from further analyses.

### SRT indexes

The results of the SRT analyses are saved into two separate csv (comma separated values) files. The first of these reports key results of the analyses on a trial-by-trial basis, including information about participant number, trial number, user-specified codes for stimulus conditions, key data used in the SRT analysis, and the result of the SRT analysis (i.e., SRT, or information that the SRT was rejected). The second csv file provides aggregated data summarizing the number of valid trials, average SRTs, and number of trials without SRTs (missing saccades) as a function of stimulus condition. If the analyses are applied for data from multiple participants, the data for separate participants are provided on a row-by-row basis in a format that can be directly read by most statistical analyses packages.

The average SRT is calculated as the mean of valid gaze shift latencies, excluding trials without gaze shifts (i.e., trials on which the gaze remains in the location of the first stimulus for the entire duration of the analysis window) and nonscorable trials that failed the postanalysis verification checks. It is noteworthy, however, that in studies with special populations, this approach can result in a number of trials being excluded from the analysis in some experimental conditions (e.g., the probability of trials without gaze shifts can be relatively high in cognitively demanding tasks or tasks involving disengagement from complex stimuli such as faces and facial expressions; Hutton, 2008; Leppänen et al., 2011). For this reason, we also added an index that includes all valid trials in the SRT analysis (i.e., trials with a gaze shift and trials without a gaze shift, excluding nonscorable trials that failed the postanalysis checks) and describes the proportion of attentional dwell-time on the first stimulus of the time window available for the saccade (i.e., the time interval from the shortest to the longest acceptable SRT). For example, in a typical paradigm with a 150- to 1,000-ms window for attention disengagement, the index would be calculated as

$$ \mathrm{SRT}\kern0.5em \mathrm{index}=\frac{{\displaystyle {\sum}_{i=1}^n\left(1-\frac{1000-{x}_i}{850}\right)}}{n}, $$

where *x*_*i*_ is the time point of saccadic eye movement on a given trial *i* (i.e., last gaze point in the area of the first stimulus preceding a saccade toward the peripheral stimulus) and *n* is the number of scorable trials in a given experimental condition. In this index, the shortest acceptable SRT (150 ms) results in 0, and the longest possible SRT (or lack of saccade, which is equal to the last measured data point at the first stimulus at 1,000 ms) results in 1.

## Results and discussion

To test the performance of the proposed approach to infant SRTs, we used data from two ongoing longitudinal studies. We used the example data for the purposes of (1) optimizing user-defined setting for a typical infant SRT paradigm, (2) comparing automatically extracted SRTs with those obtained manually from video records, (3) examining the robustness of the automated analyses against variations in calibration, number of trials, and data quality, and (4) testing the test–retest reliability of the analyses.

### Example data

The first example data consisted of infants from an ongoing longitudinal study (study 1) that began in April 2012 and consisted of laboratory assessments at 5, 7, 12, 24, and 48 months of age (Forssman et al., 2013; Kaatiala, Yrttiaho, Forssman, & Leppänen, 2013; Peltola, Hietanen, Forssman, & Leppänen, 2013). A total of 126 (55 females) infants were enrolled in the study, and all available data from the 5-month (*M* = 152.43 days, *SD* = 3.64 days) and 7-month (*M* = 213.85 days, *SD* = 4.39 days) visits were used in the present analyses, with the exception of data from one infant who was born preterm (<37 weeks). The second data set (study 2) consisted of 21 infants serving as a control group in a randomized-controlled study examining the training of attentional control in infants (Forssman, Wass, & Leppänen, 2014). Study 2 included assessments at 9 months of age (*M* = 283.63 days, *SD* = 3. 80 days) and two post-assessments at 9.5 and 11 months, respectively. All available data from study 2 were used in the present analyses. Ethical permissions for the studies were obtained from the Ethical Committee of Tampere University Hospital or Committee of Research Ethics at the University of Tampere. In both studies, an informed consent was given by the parents of the participants before the start of the study.

In the example studies, the infants sat on their parents lap at a ~60-cm viewing distance in front of a corneal-reflection eyetracker (Tobii TX300, Tobii Technology, Stockholm, Sweden), integrated with a 23-in. monitor. The monitor subtended ~46° in the *x* dimension and ~27° in the *y* dimension. Before testing, the eyetracker was calibrated by using the infant calibration procedure within the Tobii Studio software (study 1) or a custom-written MATLAB script (study 2). The calibration proceeded by showing the infant an audiovisual animation sequentially in five locations on the screen. The outcome of the calibration procedure was read from an illustration showing the offset between measured gaze points and the center of the given calibration location. If the first calibration was not successful (i.e., one or more calibrations were missing or were not properly calibrated), the calibration was repeated at least two times to attain satisfactory calibration for all five locations. If one or more calibration points were missing after >2 attempts at recalibration, the final calibration outcome was accepted, and the experiment was started. Because our study did not rely on a precise spatial tracking accuracy (see below), we found it most practical to accept all infants for the data analyses (i.e., infants with fewer than five satisfactory calibration points) but examined the potential impact of the calibration outcome on the measures of interest below. For the younger participants (i.e., 5- to 7-month-olds; study 1), attaining any successful calibration point even after several recalibration attempts was not always possible; the experiment was then run without eye tracking, and infants’ eye movements were analyzed from the video recording.

SRTs were measured by using a paradigm in which an attention-grabbing stimulus (a red circle or an animation) attracted the infant’s attention to the center of the screen. After the infant fixated the attention getter, as determined on the basis of video monitoring (study 1) or eye tracking (study 2), the trial was initiated manually by the experimenter (study 1) or automatically by a gaze-contingent script (study 2). Two stimuli were presented on each trial. The first stimulus was a picture of a face or a facelike pattern (Forssman et al., 2013) that measured ~14° of horizontal visual angle and was presented at the center of the screen for 4,000 ms. The second (a geometric shape or an animation) was presented 1,000 ms after the onset of the first stimulus on the left or right side of the screen (~14° from the center) and remained on the screen for 3,000 ms. In study 1, the second (lateral) stimulus was a geometric shape (a black-and-white checkerboard pattern or vertically aligned circles). In study 2, the lateral stimulus was an animated movie that started to play upon the infant’s first fixation (point of gaze) to the target area. The analyses of study 1 data included the first 24 trials out of a total of 48 trials (as described in Forssman et al., 2013), unless stated otherwise. The analyses of study 2 data included all 48 trials. In study 1, the test was written on E-Prime software and E-Prime extensions for Tobii (Psychology Software Tools, Inc.) interfacing with a Tobii TX-300 eyetracker. In study 2, the calibration and the disengagement script were run on custom-written MATLAB scripts, Psychtoolbox, and the Talk2Tobii toolbox,^1^ interfacing with a Tobii TX-300 eyetracker.

### User-defined parameters for SRT analyses

On the basis of the iterative analysis of a subsample of participants from study 1 (*n* = 15), the user-defined parameters were set as follows. (1) The minimum duration for the first stimulus prior to the presentation of the second stimulus was 900 ms, the maximum duration 1,100 ms, and the minimum duration for the second stimulus 1,000 ms.^2^ (2) A 37-sample median filter was used to filter the data, equaling 123 ms for data sample at 300 Hz; this median filter was considered sufficient to remove technical artifacts without losing important data such as saccades that typically take 100–130 ms to program (Inhoff & Radach, 1998; Radach, Heller, & Inhoff, 1999). (3) Data with validity codes 0 and 1 were accepted as valid points of gaze (cf. Tobii TX-300 user manual); all data with validity codes 2 or higher were interpolated. (4) The threshold for saccade (i.e., *x*-coordinate value that was used to detect eye movements away from the location of the first stimulus) was set at 30 % from the edges; this threshold, including a ~2.7° margin on both sides of the face image, was capable of detecting 75 out of 76 target-directed saccades in the test subsample without resulting in false positives or underestimation of saccade latencies. (5) The threshold for the longest interpolated (nonvalid) segment was set to 200 ms; this criterion helped to retain data in the analysis while also not resulting in an unacceptable risk of false negatives (i.e., if the period of interpolation is sufficiently long, the likelihood that gaze transitions from the first stimulus to the second stimulus and back [i.e., 1st–2nd–1st] take place during the interpolation period, resulting in false negative for saccades). (6) The minimum fixation for the first stimulus prior to fixation was set at .70 of the total possible gaze samples available during the presentation window (including interpolated data). (7) The minimum and maximum accepted disengagement times were set at 150 and 1,000 ms, respectively (Forssman et al., 2013; Leppänen et al., 2011).

### Percentage of valid SRTs

Of the initial data from study 1, the analyses of SRTs at 5 months of age were performed for 95 infants who had data available. For the remaining infants in the sample, data were missing for various reasons, including delayed enrollment to the study (*n* = 7) and technical difficulties/fussiness (*n* = 23). The analyses of SRTs at 7 months were conducted for 118 participants. Data for the remaining participants were missing because of dropouts (*n* = 2) or technical difficulties/fussiness (*n* = 5). For the analysis of the 5-month data, valid SRTs were obtained for 68.3 % of trials. For the analysis of 7-month data, valid SRTs were obtained for 79.4 % of the trials. For study 2, the percentage of valid trials was 73.2 % for the 9-month assessment, 74.0 %, for the 9.5-month assessment, and 71.8 % for the 11-month assessment.

### Comparisons of automatically versus manually extracted SRTs

**Fig. 3:**
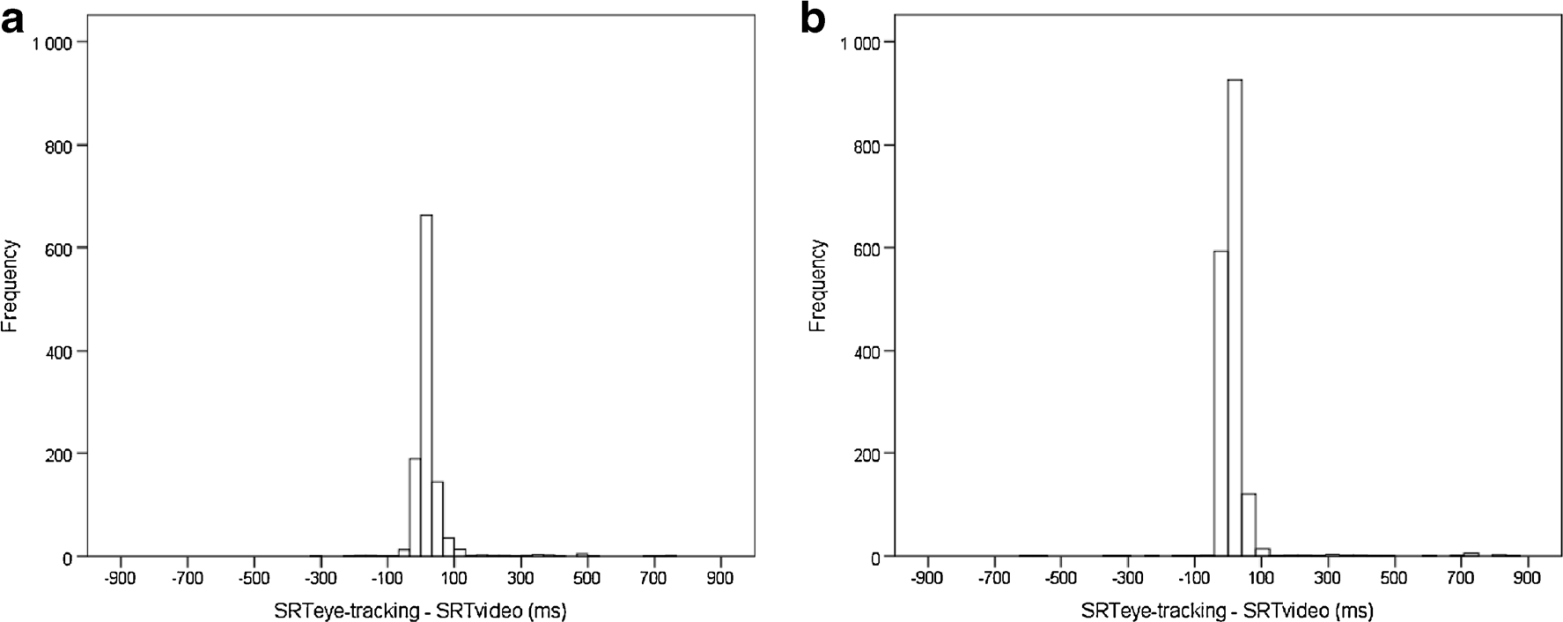
Histograms showing the distribution of difference values between automatically and manually coded saccadic reaction times (i.e., SRT_eye-tracking_ − SRT_video_) for all trials in the 5- (**a**) and 7-month (**b**) assessments

To validate the proposed eye-tracking approach for the analysis of SRTs, we compared the automatically extracted SRTs with those obtained manually from video records of participants’ eye movements, using data from study 1. A coder who was blind to the stimulus condition coded saccadic eye movements from the videos by using a frame-by-frame (30 frames per second) playback. The comparisons of eye-tracking and video data were conducted on a trial-by-trial basis using data from trials with a valid SRT (or a value of 1,000 ms indicating a missing gaze shift) in both data sets (Fig. 3). For the 5-month assessments, a total of 1,097 trials with overlapping eye-tracking and video data were available. The temporal discrepancy between the automatically and manually obtained SRTs was < 100 ms for 1,046 out of 1,097 trials (95.4 %; mean difference, 24.1 ms; median, 13.2; 95 % CI, 18.2–28.9). For the 7-month assessments, 1,690 trials with overlapping eye-tracking and video data were available. The temporal discrepancy between the automatically and manually obtained SRTs was <100 ms for 1,648 out of 1,690 trials (97.5 %; mean difference, 20.3 ms; median, 10.0; 95 % CI, 14.5–25.4). These results are in accordance with the results of a previous study examining the correspondence of automatic and manually coded saccades in a different paradigm (Shukla, Wen, White, & Aslin, 2011).

The relatively rare cases of large (>100-ms) discrepancy values between automated and manual SRT analyses (2.5 %–4.6 % of trials) consist mostly of trials on which the infant’s saccade to the lateral distractor was completed in two phases (i.e., the first movement close to the edge of the area of the first stimulus was followed by a second eye movement toward the target), and the eye-tracking and video-based analyses detected the onset of the saccade at different points in time. Other reasons for larger discrepancies included apparent false positives in manual coding, as well as other technical or unknown reasons. Examples of the typical trials resulting in larger discrepancy are shown in Supplementary Fig. 2.

### Sensitivity to calibration outcome and number of valid trials

In studies with poorly cooperating participants, the outcome of the calibration procedure and the number of trials available for analyses can vary substantially between participants. To examine whether the proposed method of SRT analysis is robust against problems in calibration, we used data from the 5-month visit (study 1) as variations in calibration tended to be highest in this data set. We examined whether the trial-by-trial error associated with automated SRT calculation, as assessed by the difference in automatically and manually detected SRTs, was higher in infants with one or more missing calibration points (33.5 % of participants). This analysis showed, as compared with the whole-sample analyses reported above, that the proportion of >100-ms errors was only slightly higher in the subsample with poor calibration (i.e., 4.6 % in the whole sample vs. 5.6 % in the subsample with incomplete calibration). To examine whether there is any systematic association of the SRTs with the number of valid trials available for analysis, we used data from all 48 trials in studies 1 and 2 to calculate correlations between the stimulus condition-specific average SRTs and the number of valid trials available for analysis (range: 3.5–12 and 3.6–16 per condition in the example studies 1 and 2, respectively).^3^ The correlations (Pearson’s *r*) were low and not significant for all comparisons [5 months, *r*(74) = −.21–.15, *p*s > .05; 7 months, *r*(103) = −.18–.03, *p*s > .05; and 9 months, *r*(19) = −.37–.02, *p*s > .05]. These results suggest that there is no direct relationship between the SRTs as indexed here and the number of accepted trials.

### Sensitivity to variations in data quality

We also examined whether the accuracy of the SRT analysis was associated with two indices of data quality: (1) precision (i.e., the degree to which reporting of the position of gaze is consistent between samples) and (2) robustness (i.e., how broken or fragmented contact is with the eyetracker during recording). The analyses were performed using data from the 5-month visit in study 1.

In order to examine data quality, eye-tracking data segments were excerpted either for the period between the start of each trial and the time of first saccadic eye movement (as coded using the proposed algorithms) or for instances in which no disengagement was recorded, the first 2,000 ms of the trial. Precision was calculated using the algorithms described in Wass et al. (2014). Robustness was previously calculated as the mean duration of usable data fragments (Wass et al., 2014). However, this was not considered optimal in the present instance, since the duration of data segments entered into the analysis was variable; instead, we estimated robustness by calculating the proportion of unavailable data within each trial (following, e.g., Holmqvist et al., 2011).

**Fig. 4:**
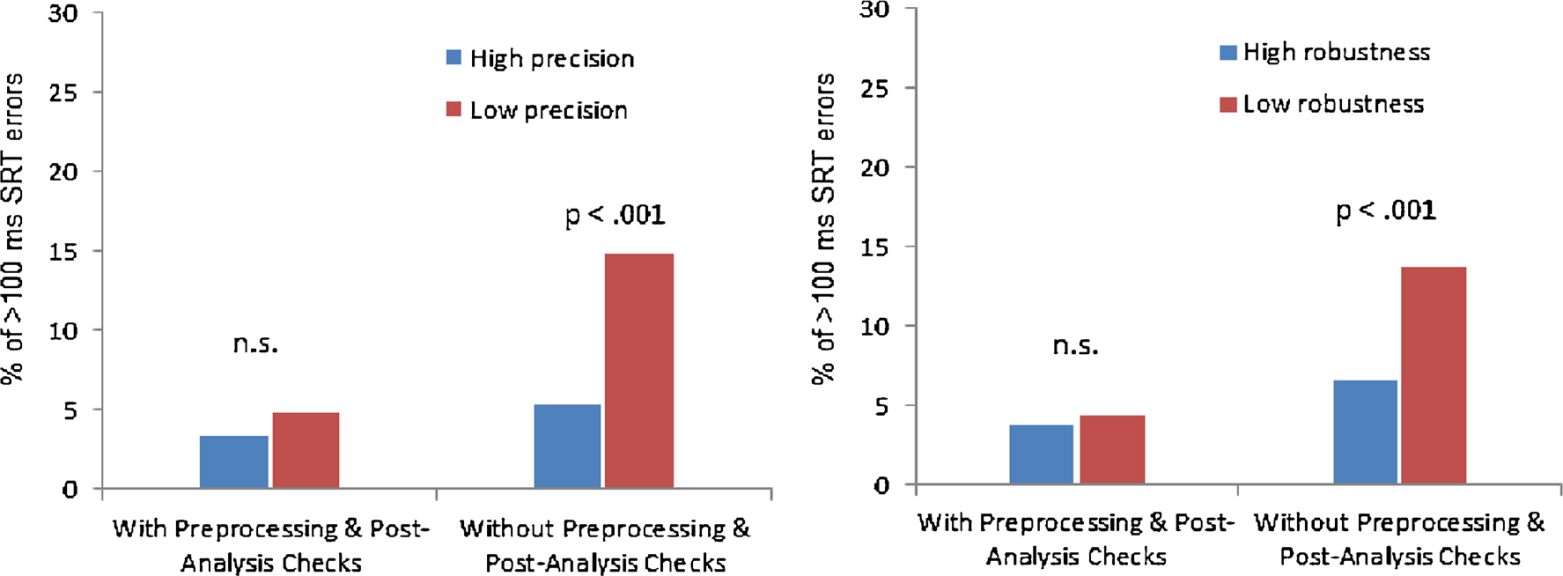
Percentage of trials with large (>100-ms) saccadic reaction time errors in analyses with the proposed preprocessing routines, 2.7° margins on the sides of the first image, and postanalysis checks versus analyses without the preprocessing routines, widened margins, and postanalysis checks. The percentages are presented separately for trials with low versus high data quality based on median splits of data precision and robustness indices

To examine whether the accuracy of the SRT analysis (i.e., the difference in the eye-tracking and video-based coding) differed between trials with high- versus Low-quality data, we used median splits to divide the trial-by-trial data into trials with high versus low precision and trials with high versus low robustness. We then examined whether the number of trials with large SRT errors (>100-ms difference in automatic vs. manual coding) differed significantly between the trial groups by using Pearson’s chi-square test. We chose to examine the number of large SRT errors, instead of mean SRT error values, because of the limited temporal resolution of the video coding. The results showed that the number of large SRT errors was generally low (3.3 %–4.9 %) in the analyses conducted with the new routines and user-defined settings and that these numbers did not differ between trials with high versus low precision (*p* = .19) or between trials with high versus low robustness (*p* = 26; Fig. 4).

We next recalculated the SRTs in our example data by using a “typical” approach without the modifications we have incorporated in this article and examined whether the accuracy of these analyses was associated with data quality (as has previously been reported by Wass, Forssman, et al., 2014). This analysis was also aimed at establishing the importance of the proposed pre- and postanalysis routines and criteria in the SRT analysis. The typical analysis was performed without applying the proposed preprocessing and postanalysis verification routines and with narrower margins on the sides of the first image (i.e., 1° instead of 2.7°). The trial-by-trial error in the SRT calculation (i.e., eye tracking − video) and the parameters reflecting data quality were calculated as described above. Results suggested that there was a significant relationship between the number of >100-ms SRT errors and data precision, χ^2^ = 28.5, *p* < .001, *R* ^2^ = .03, and between the number of >100-ms SRT errors and data robustness, χ^2^ = 15.8, *p* < .001, *R* ^2^ = .01. As is shown in Fig. 4, the number of large SRT errors was notably higher when the typical approach without the pre- and postanalysis routines was used to analyze trials with less precise or robust data. Together, these results indicate that the proposed preprocessing and postanalysis check routines are particularly important in analyzing SRTs from low-quality data.

### Test–retest reliability

**Fig. 5:**
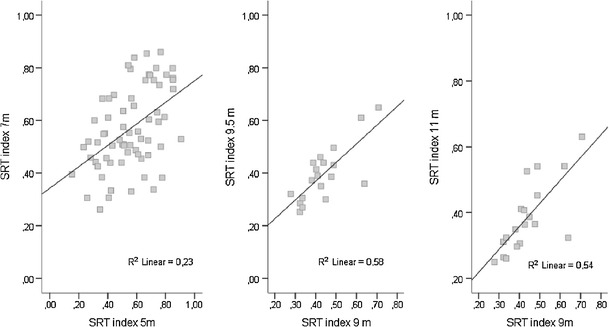
Longitudinal association of saccadic reaction times (SRTs) measured from the same infants at 5 and 7 months and at 9, 9.5, and 11 months

Previous longitudinal research (Hunnius et al., 2006) has shown that disengagement undergoes a relatively rapid developmental course (i.e., age-related increase in frequency and decrease in latency) during the first months of life and that this development appears to stabilize at 5–6 months of age. Given these findings, we expected stability in the SRTs over time in the age range studied in the example data set. When all 48 trials in both studies were included in the analyses (and after excluding participants with < 3 trials per experimental condition), longitudinal data were available for 68 infants at 5 and 7 months (study 1) and 19 infants from 9, 9.5, and 11 months of age (study 2). The test–retest correlations of overall mean SRT indices are shown in Fig. 5. The SRT index was only moderately correlated between 5 and 7 months, *r*(68) = .48, *p* < .001, *R* ^2^ = .23, but appeared to become more stable between 9, 9.5, and 11 months of age, *r*s(19) = .74 and .80, *p*s < .001, *R* ^2^ = .54 and .58. These analyses with the present routines and metrics compare favorably with results from Wass and Smith (2014), who reported test–retest reliability of *r*(20) = .37, *p* = .09 on SRTs obtained from typical 11-month-olds during presentation of a noncompetition disengagement task.

## Conclusion

In this report, we have demonstrated that when applied with proper preprocessing and data quality checks, standardized and automated computer routines can be applied for the analysis of SRTs from eye-tracking data collected from poorly cooperating participants. Our analyses also demonstrated that the SRT index introduced in this study has moderate stability in infancy, supporting the utility of this metric in quantifying individual infant performance. It is important to note, however, the overall success of the eye-tracking analysis continues to be a challenge (i.e., percentage of data retained for final analysis), especially with younger infants. Also, an important limitation of the present approach was that the temporal accuracy of the SRT analysis was evaluated against low-resolution video data (30 fps). These limitations notwithstanding, the present data provide support for the use of SRTs as an accessible, objective, and widely applicable marker to examine neurocognitive function in a variety of populations (Bar-Haim, 2010; Bar-Haim, Morag, & Glickman, 2011; Chawarska et al., 2010; Elison et al., 2013; Elsabbagh et al., 2009; Forssman et al., 2013; Hunnius et al., 2008; Scerif et al., 2005).

## Electronic supplementary material

Below is the link to the electronic supplementary material.

ESM 1 (DOCX 71 kb)

ESM 2 (DOCX 40 kb)

ESM 3 (DOCX 126 kb)

## Footnotes

1
http://psy.ck.sissa.it/t2t/About_T2T.html

2 This criterion was used to detect rare cases in which the software and hardware failed to present the stimuli (or collect gaze data) for the required duration.

3 Consistent with the criteria used in previous studies (e.g., Forssman et al., 2013), participants with three or more valid trials per condition were included in the analysis.
