# Supplementary material for: Widely applicable MATLAB routines for automated analysis of saccadic reaction times
Source: Behav Res Methods. 2014 May 2;47(2):538–48. doi: 10.3758/s13428-014-0473-z (PMC4427653; doi:10.3758/s13428-014-0473-z)
Supplement: Supplementary file 1 — (DOCX 71 kb) [file 13428_2014_473_MOESM1_ESM.docx]

Supplementary Figure 1 for Leppänen et al. *Widely applicable MATLAB routines for automated analysis of saccadic reaction times*

**
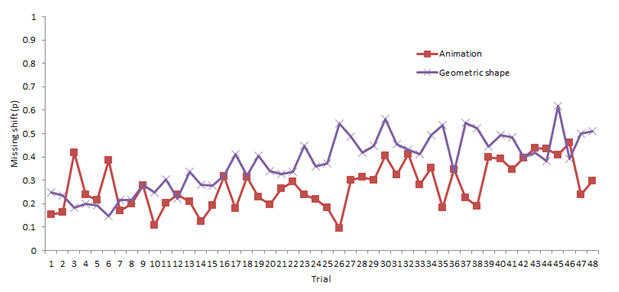
**

Proportion of trials with a saccadic eye movement from the first stimulus at the center of the screen (a picture of a face or face-like pattern) to a second stimulus in the periphery (a static geometric shape or a dynamic, gaze-contingent animation) in 7 months old infants. The proportions were calculated as the number of infants who made a saccade out of all infants with valid data for the given trials (Max Ns = 103 and 87 in the static and dynamic conditions, respectively). The data demonstrate that there in an increase in number of missing saccades over the course of the experiment in the static condition, whereas the saccade rate remains reasonably steady in the dynamic, gaze-contingent animation condition (i.e., the animations starts to play when the infant’s point of gaze reaches the area of the animation).
