# Supplementary material for: Widely applicable MATLAB routines for automated analysis of saccadic reaction times
Source: Behav Res Methods. 2014 May 2;47(2):538–48. doi: 10.3758/s13428-014-0473-z (PMC4427653; doi:10.3758/s13428-014-0473-z)
Supplement: Supplementary file 2 — (DOCX 40 kb) [file 13428_2014_473_MOESM2_ESM.docx]

Supplementary Figure S2 for Leppänen et al. *Widely applicable MATLAB routines for automated analysis of saccadic reaction times*

Samples of eyetracking data were collected during presentation of a sequence learning task to typical 12-month-old infants. Stimuli were presented on a Tobii TX300 and the Matlab SDK under optimum lab conditions (even top-lighting, illumination c. 300 lux, black surrounds)^[[1]](#footnote-1)^. 44 15-second trials were analysed (11 infants, 4 trials per infant). a) histogram of proportion of data samples missing. On average 18% of all available data samples were missing (std 18). b) a histogram showing the durations of usable data segments obtained. The average usable data segment obtained was 1.15 seconds (std .98).

1. <http://www.tobii.com/Global/Analysis/Downloads/User_Manuals_and_Guides/Tobii_TX300_EyeTracker_UserManual.pdf> [↑](#footnote-ref-1)
