# Supplementary material for: Widely applicable MATLAB routines for automated analysis of saccadic reaction times
Source: Behav Res Methods. 2014 May 2;47(2):538–48. doi: 10.3758/s13428-014-0473-z (PMC4427653; doi:10.3758/s13428-014-0473-z)
Supplement: Supplementary file 3 — (DOCX 126 kb) [file 13428_2014_473_MOESM3_ESM.docx]

Supplementary Figure 3 for Leppänen et al. *Automated analysis of saccadic reaction times in human infants.* Examples of the relative rare trials with >100 ms discrepancy in automatically and manually extracted saccadic reaction times (SRTs).

**
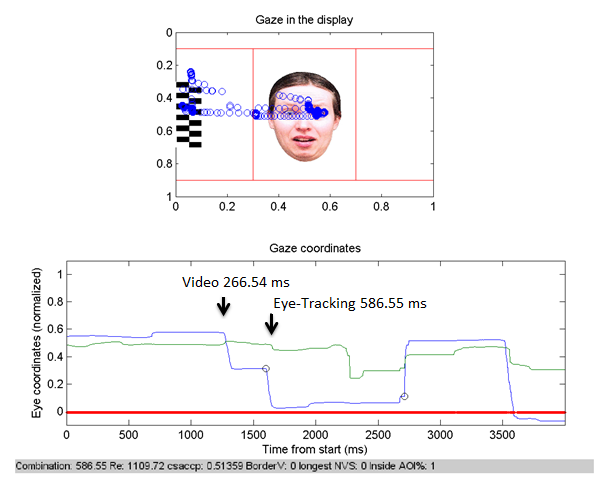
**

**Example 1**. The upper part of the graph shows gaze points for the entire duration of the trial (4 sec) superimposed on the stimulus display. The lower part shows the x (blue) and y (green) coordinates of the eyes as a function of time. For this trial, the SRT was detected earlier in the manual analysis of eye movements from the video (266.5 ms) than in the automated analysis of eye-tracking data (586.6 ms). The discrepancy is explained by the “phased” nature of the saccade; that is, the gaze shifts to the edge of the face area (this shift is detected in the video analysis) before crossing the border of the face area and moving to the target area (only this latter shift is detected in the eye-tracking analysis).

**
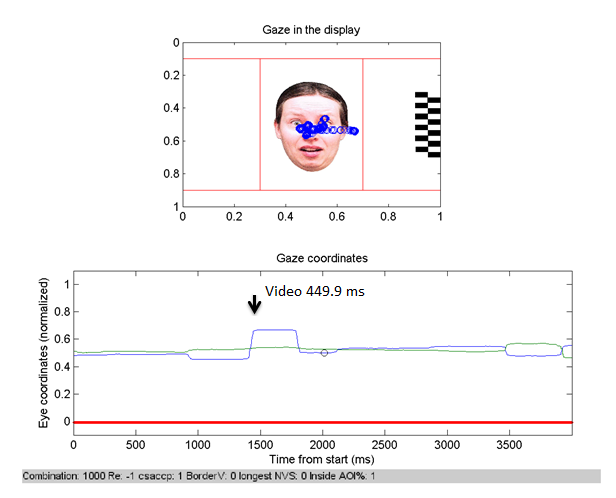
**

**Example 2**. Potential false positive in the video analysis. The saccade is below threshold in the eye-tracking analysis (no SRT is returned) and above threshold in the video analysis (SRT is coded at 449.9 ms post-stimulus).
